# A second species of *Tyloceridius* Malaise (Hymenoptera, Tenthredinidae)

**DOI:** 10.3897/zookeys.396.6566

**Published:** 2014-04-02

**Authors:** Meicai Wei, Gengyun Niu, Andreas Taeger

**Affiliations:** 1 Key Laboratory of Cultivation and Protection for Non–Wood Forest Trees (Central South University of Forestry and Technology), Ministry of Education, Changsha 410004, China; 2 Laboratory of Insect Systematics and Evolutionary Biology, Central South University of Forestry and Technology, Changsha, Hunan, 410004, China; 3 Senckenberg Deutsches Entomologisches Institut, Eberswalder Str. 90, 15374 Müncheberg, Germany

**Keywords:** Symphyta, Tenthredininae, China, Nepal, new species, identification key

## Abstract

*Tyloceridius* Malaise, 1945 and *T. dorsatus* (Mocsáry, 1883) are redescribed. *Tyloceridius stictocephalus* **sp. n.** from China and Nepal is described. *Tyloceridius* is recorded in China for the first time.

## Introduction

Malaise (1945) described the monotypic *Tyloceridius* from Kashmir and the Himalaya and designated *Allantus dorsatus* Mocsáry, 1883 as the type species, with *Rhogogastera bituberculata* Cameron, 1906 placed as a junior synonym. Since then, Muche (1983) recorded this species from India (Uttaranchal), and Togashi (1989) from Pakistan (Islamabad). Saini et al. (2006) listed the provinces Himachal Pradesh, Jammu and Kashmir, and Uttaranchal for the distribution in India. Saini (2007) redescribed the genus and both sexes of the species and illustrated the lancet, penis valve and gonoforceps. No further species of the genus was hitherto known (Taeger et al. 2010).

During a survey of the sawfly fauna of Tibet in the summer of 2003, a specimen of *Tyloceridius* was collected at Yadong. The examination of the lectotype of *Tyloceridius dorsatus* showed that the specimen from Tibet belongs to an undescribed species. Later, a series of the new taxon from Nepal was found in unidentified material at the SDEI.

## Material and methods

Terminology of sawfly genitalia follows Ross (1945). Wing venation follows Niu and Wei (2010, Plate 1).

The images were obtained using a Leica S8APO digital camera and Motic BA400 microscope and further processed with Helicon Focus 5.1(©HeliconSoft) and Adobe Photoshop CS6 software. The images based on specimens from Nepal were taken at the DEI with a Leica DFC 495 digital camera and M205 C microscope and processed with CombineZ5.3 and PhotoImpact X3.

Abbreviations used are: OOL = distance between the eye and outer edge of lateral ocellus; POL = distance between the mesal edges of the lateral ocelli; OCL = distance between a lateral ocellus and the occipital carina or hind margin of the head; ED = the ratio of the distance between anterior-lower corner of eyes and the greatest diameter of an eye. CSCS = Central South University of Forestry and Technology, Changsha, P. R. China;
SDEI = Senckenberg Deutsches Entomologisches Institut, Müncheberg, Germany; HNHM = Hungarian Natural History Museum, Budapest, Hungary; NHRS = Naturhistoriska riksmuseet, Stockholm, Sweden; NKME = Naturkundemuseum Erfurt, Germany; USNM = National Museum of Natural History, Washington D.C., USA.

## Taxonomy

### Tyloceridius

Malaise, 1945

http://species-id.net/wiki/Tyloceridius

Tyloceridius Malaise, 1945: 171. Type species: *Allantus dorsatus* Mocsáry, 1883, by original designation.

#### Diagnosis.

Distinguished from other genera of Tenthredininae by deep furrow on ventral side of antennal flagellum and the very large, protruding and isolated supra-antennal tubercles.

#### Description.

**Figures 1–7.**
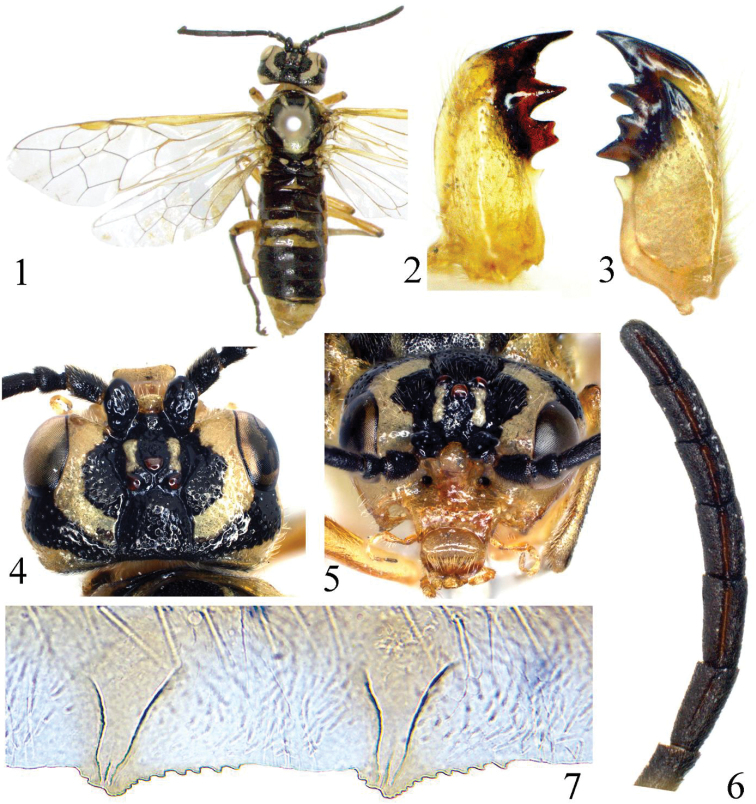
*Tyloceridius stictocephalus* sp. n., holotype, female **1** Adult, dorsal view **2** Left mandible **3** Right mandible **4** Head, dorsal view **5** Head, frontal view **6** Antenna 7 The 5^th^ and 6^th^ serrulae of lancet.

**Figures 8–10.**
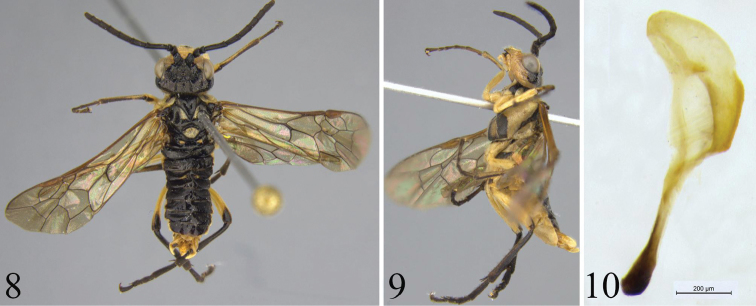
*Tyloceridius stictocephalus* sp. n., paratype, male **8** Adult, dorsal view **9** Adult, lateral view **10** Penis valve (scale bar = 200 μm).

**Figures 11–15.**
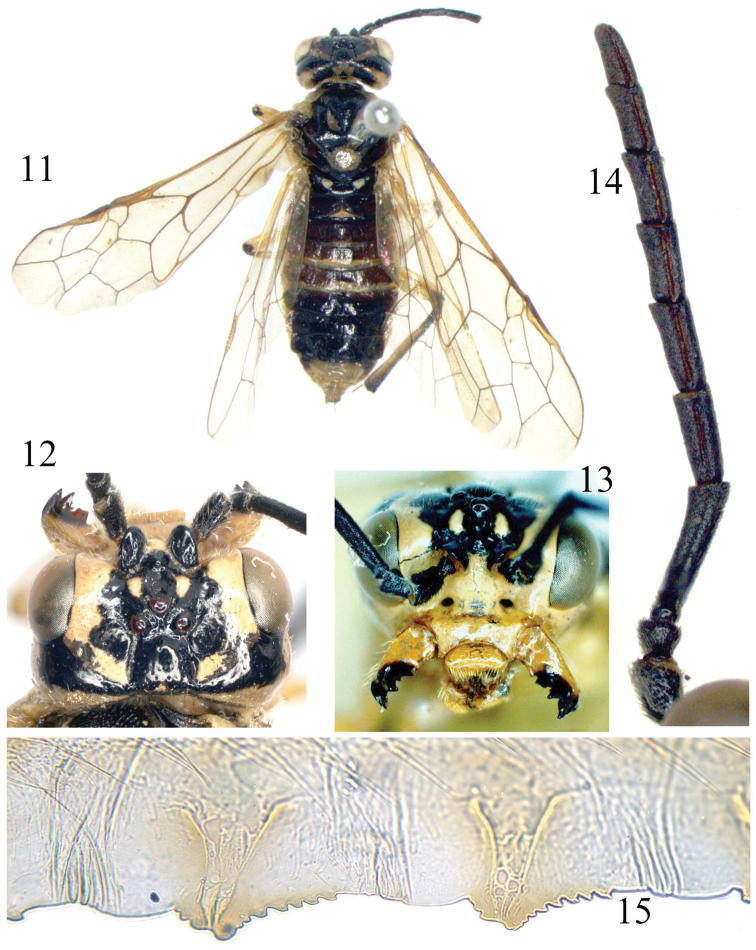
*Tyloceridius dorsatus* (Mocsáry, 1883), female **11** Adult, dorsal view **12** Head, dorsal view **13** Head, frontal view **14** Antenna **15** The 5^th^ and 6^th^ serrulae of lancet **11** lectotype **12–15** a specimen from North India (SDEI).

Body robust (Figs 1, 8). Clypeus transversely subconvex at basal third, anterior margin sharp, quarter-circularly incised with acute lateral teeth; apical third of labrum deflexed, and labrum thus truncate at apex in front view (Figs 5, 11); mandibles strongly bent at apical third, asymmetric (Figs 2, 3), right one with 1 basal tooth, 3 inner teeth; one of them placed on the dorsal side (Fig. 3); left mandible with 1 basal tooth and 2 inner teeth, without dorsal tooth (Fig. 2); malar space longer than diameter of median ocellus; inner margins of eyes very feebly S-curved and converging downwards, distance between eyes much longer than height of eye (Figs 5, 11); supra-antennal tubercles large, highly elevated and quite free-standing (Figs 4, 10, 12); head elongated behind eyes, occipital carina low but distinct; interocellar furrow broad and deep, postocellar furrow broad and shallow. Antennae stout and uniformly thick; 2^nd^ antennomere about as long as broad, flagellum with a very deep and sharp longitudinal furrow along the outer-side from apex of the 3^rd^ antennomere to 9^th^ antennomere, 3^rd^ antennomere distinctly longer than 4^th^ (Figs 6, 9). Anterior lobe of pronotum broad, without marginal carina, with broadest part about 3 × diameter of an ocellus; ventral margins of propleura narrowly but distinctly meeting; mesoscutellum flattened, mesoscutellar appendage with an obtuse middle ridge. anterior basin of metascutellum narrow, furrow like; dorsal lobe of metepimeron long and linear; mesosternal thorns wanting; posterior corner of metepimeron round, without appendage. Basal plates (first tergite) of abdomen contiguous on meson, without large membranous blotch (Fig. 8). Inner tibial spur of fore leg bifurcate at apex; hind coxa small, hind femur not reaching apex of abdomen, distinctly shorter than hind tibia, hind tibial spur shorter than half length of metabasitarsus; metabasitarsus about as long as or slightly longer than following 3 tarsomeres together; claw cleft, inner tooth shorter than outer tooth. Venation similar to *Tenthredo*, anal cell of forewing with a short and erect cross vein at basal 2/5; hind wing with 2 closed middle cells, anal cell sessile or shortly petiolate (Figs 1, 8).

#### Distribution.

Himalaya: China (Tibet), India (Jammu & Kashmir, Uttaranchal, Himachal Pradesh), Nepal, Pakistan (Islamabad).

#### Key to species

**Table:** 

1	Dorsal side of head strongly shiny, with few widely scattered punctures; postocellar area not distinctly more strongly punctured than lateral parts of vertex; in both sexes, posterior yellow margins of tergites 3–4 not broader medially than laterally, usually connected with lateral yellow band of abdomen	*Tyloceridius dorsatus* (Mocsáry, 1883)
–	Dorsal side of head usually strongly punctured, distance between punctures about 1–3 × diameter of punctures; if lateral parts of vertex rather smooth, postocellar area more densely and distinctly more strongly punctured than lateral parts of the vertex; in female, posterior yellow margins of tergites 3–4 broader medially than laterally, not connected with lateral yellow band of abdomen; in male, tergites 3–4 dorsally completely black or posterior yellow margins less developed than in female	*Tyloceridius stictocephalus* sp. n.

### Tyloceridius stictocephalus sp. n.

http://zoobank.org/49ED98B4-BA75-4E93-9CC4-68B8DDA26C44

http://species-id.net/wiki/Tyloceridius_stictocephalus

Figs 1 –10

#### Description.

Holotype ♀ (Fig. 1).

Body length 11 mm. Greenish yellow, following parts black: apex of each mandible; antennae entirely; supra-antennal tubercle, frons and adjacent area of upper inner orbit except a stripe on frontal ridge, postocellar area, a broad band from upper hind corner of each eye to postocellar area (Figs 1, 4); a medial band on pronotum; meso- and metanotum, except mesoscutellum and 2 small triangular spots on mesoscutal lateral lobe and 2 linear spots on mesoscutal median lobe; abdomen above, except for tergites 8–10 and a broad band on posterior margin of tergites 3–4 (in the middle covering about two thirds, laterally about one third of the length of the tergite); depressed upper corner of mesepimeron and a minute and roundish dot in lower posterior corner of mesepisternum. Legs greenish yellow, following parts black: a small dot on extreme apex of each femur; a narrow dorsal stripe on each tibia and tarsus. Wings hyaline with very faint yellow tinge, vein C and stigma yellow brown, other veins pale brown. Body hairs silver.

Body shiny; labrum without large punctures; central area of clypeus, outer sides of mandibles with some large punctures, head above antenna including supra-antennal tubercle with large and deep punctures, interspaces between punctures about as large as a puncture, surface smooth and shiny; punctures on postorbit sparser but distinct; punctures on mesoscutal median lobe and mesoscutal lateral lobe smaller than punctures on head, interspaces shiny; posterior margin of mesoscutellum densely punctured, dorsal side of mesoscutellum hardly punctured; mesoscutellar appendage impunctate, parapsis of mesothorax microsculptured; metascutellum with some minute punctures; mesepisternum densely punctured, narrow interspaces smooth, strongly shiny; metepisternum densely punctured, mesepimeron and metepimeron polished, with scattered punctures. Abdominal tergites 1–2 strongly shiny, tergite 3 weakly microsculptured in basal half, tergites 4–7 distinctly microsculptured and punctured in basal half.

Clypeus and labrum as Fig. 5; left mandible as Fig. 2, right mandible as Fig. 3; ED = 1.7; supra-antennal tubercle larger than scape (Fig. 4), clearly above top of ocelli in lateral view; a distinct tubercle present on anterior margin of middle fovea and almost in line with anterior margins of supra-antennal tubercles; frontal walls broad and flat, parallel to each other; postocellar area 1.2 × as broad as long; lateral furrows deep, distinctly divergent backwards; POL : OOL : OCL = 1 : 2.5 : 2.5; head behind eyes about as long as eyes in dorsal view, lateral edge roundly curved (Fig. 4); occipital carina distinct in entire length. Antennae stout, weakly compressed, 0.9 × length of head and thorax combined, antennomere 3 0.9 × length of antennomeres 4 and 5 combined, antennomeres 6–8 1.7 × as long as broad (Fig. 6). Anal cell of hind wing sessile. Ovipositor sheath 1.1 × length of fore tibia. Lancet with 21 serrulae, serrulae 5–6 as Fig. 7.

Variability (females). Body length 10–11 mm. Punctation on upper head varies from sparse to dense; supra-antennal tubercle sometimes only with few punctures and / or smaller than scape; tergites 1–2 smooth to rather densely punctured laterally and on basal half; POL : OOL : OCL = 1 : 2.0–2.7 : 2.0–2.6; postocellar area 1.2–1.4 × as broad as long; head behind eyes 0.8–1.0 × as long as eyes in dorsal view. Ratio of length and breadth of antennomeres 6–8: 1.5–1.9. Black spot on lower mesepisternum may be indistinct or missing; pale color on tergite 4 may be reduced to a triangular spot; greenish color may alter (in dried specimens) to yellow.

Male. (Figs 8–10) Body length 8–9 mm. Color and structure similar to female. Generally darker than the female: pale stripe on upper inner orbit very narrow and only exceptionally nearly extending toward postocellar area; frontal ridges black or pale marked; hind tibia and tarsus entirely black; pale macula on mesoscutellum more or less reduced; mesoscutal median lobe usually entirely black, without pale lateral stripe; tegula black, basally pale; lower mesepisternum usually with broad black stripe, sometimes anteriorly reduced to a large spot; tergites 3-4 dorsally usually only with faintly indicated pale hind margin, sometimes completely black; subgenital plate apical margin rounded. Penis valve Fig. 10.

Pictures of several paratypes are given in original resolution at http://dx.doi.org/10.6084/m9.figshare.781286

#### Etymology.

The specific epithet, an adjective, refers to the distinctly punctured head.

#### Distribution.

China (Tibet), Nepal (Karnali).

#### Holotype.

♀, **China:** Tibet, Yadong, Naiduilashan, 27°24'51"N, 88°56'08"E, 3100m, 2003.VIII.29, Wei Meicai leg. Coll. CSCS.

#### Paratypes.

(7 ♀♀, 10 ♂♂, DEI-GISHym 17227–17243, in coll. CSCS, DEI, NHRS, NKME). Nepal, Prov. Karnali: 1 ♀, Umg. Churta 3000–3400 m, 31.V.2007 (DEI-GISHym 17228, BOLD:ACG2198); 1 ♀, Umg. Churta E Hochtal 3500–4000 m, 02.VI.–04.VI.2007; 4 ♂♂, Umg. Churta E Hochtal 3500–3800 m, 02.VI.2007; 1 ♀, Gothichaur, 29°12'10"N, 82°18'56"E, 2850 m, 09.VI.1997; 1 ♀, Gothichaur, Thymian-Wiese, 3100 m, 26.V.2007; 1 ♀ 2 ♂♂, Gothichaur, 29°11'54"N, 82°18'36"E, 2850 m, Sumpfwiese, 26.V.–05.VI.2007; 4 ♂♂, Gothichaur, 29°11'54"N, 82°18'36"E, 2850 m, Umg. Lager, 26.V.–05.VI.2007; all specimens F. Creutzburg leg.; 1 ♀, Gothichaur 2 km W, 2700 m; 20.-21.V.1995, M. Hartmann leg.; 1 ♀, Gothichaur, Wald, 29°12'10"N, 82°18'56"E, 2850 m, 08.VI.1997, M. Hartmann leg.

### Tyloceridius dorsatus

(Mocsáry, 1883)

http://species-id.net/wiki/Tyloceridius_dorsatus

Figs 11–15

Allantus dorsatus Mocsáry, 1883: 4, ♀, India orientalis. Taeger 1991: 76, lectotype designation.
Tenthredo aliena Enslin, 1912: 103. New name for *Allantus dorsatus* Mocsáry, 1883.
Tyloceridius dorsatus : Malaise 1945: 171.
Rhogogastera bituberculata Cameron, 1906: 289 [sex not given], Kashmir at 6000 ft. Synonymy by Malaise 1945: 171.

#### Description.

Lectotype of *Allantus dorsatus* ♀ (Fig. 11), additions based on other specimens are given in brackets [ ].

Body length 11 mm. Greenish yellow, following parts black: apex of each mandible; antennae entirely (Fig. 14); supra-antennal tubercle, frons and adjacent area of upper inner orbit except a short stripe on frontal ridge, postocellar area, a broad band from upper hind corner of each eye to postocellar area (Figs 12, 13); a medial band on pronotum; meso- and metanotum, except mesoscutellum and 2 small triangular spots on mesoscutal lateral lobe and 2 linear spots on mesoscutal median lobe (the latter sometimes absent); abdomen above, except for tergites 8–10 and posterior 2/5 of tergites 3–4; depressed upper corner of mesepimeron and a small and roundish spot in lower posterior corner of mesepisternum. Legs greenish yellow, following parts black: a small dot on extreme apex of each femur; a narrow dorsal stripe on fore and middle tibiae, dorsal side of hind tibia; a narrow stripe on fore and middle tarsi above (sometimes interrupted), hind tarsus entirely. Wings hyaline with faint yellowish tinge, veins and stigma pale brown, base of stigma slightly darkened. Body hairs silver.

Body shiny; labrum and outer side of mandible with some large punctures, head otherwise almost impunctate, frontal basin feebly microsculptured; mesoscutal median lobe and mesoscutal lateral lobe sparsely punctured, interspaces smooth and shiny; posterior margin of mesoscutellum densely punctured, dorsal side of mesoscutellum hardly punctured; mesoscutellar appendage and parapsis impunctate; metascutellum with some minute punctures; mesepisternum shallowly but distinctly punctured, surface smooth, strongly shiny; metepisternum densely punctured, mesepimeron and metepimeron polished, with scattered punctures. Abdominal tergites 1–2 strongly shiny, tergite 3 weakly microsculptured in basal half, tergites 4–7 distinctly microsculptured and punctured in basal half.

Clypeus, labrum, mandibles as Fig. 11; ED = 1.3; supra-antennal tubercle distinctly smaller than scape (Figs 12, 13), clearly lower than top of ocelli in lateral view; a small tubercle present in bottom of middle fovea and in line with posterior margins of supra-antennal tubercles; frontal walls broad and flat, weakly divergent forwards; postocellar area broader than long as 7 : 5; lateral furrows deep, weakly divergent backwards; POL : OOL : OCL = 15 : 29 : 27; head behind eyes about 0.7 × length of eyes in dorsal view, indistinctly narrowed (Fig. 12); occipital carina distinct in lower 2/3 and rather weak in dorsal 1/3. Antennae weakly compressed, as long as head and thorax combined, antennomere 3 0.9 × length of antennomeres 4 and 5 together, [antennomeres 6–8 1.5–2.4 × as long as broad (Fig. 14)]. Anal cell of hind wing with a very short petiole. Ovipositor sheath as long as fore tibia. [Lancet with 20 serrulae, serrulae 5–6 as Fig. 15].

Male. Body length 8 mm. Similar to female except head distinctly narrowed behind eyes in dorsal view, malar space slightly longer than diameter of median ocellus; pale stripe from upper inner orbit to postocellar area missing; subgenital plate rounded at apex; penis valve simple, valviceps weakly bent; harpe about 2 × as long as broad (see figs 678 and 680 in Saini 2007).

#### Distribution.

India: Himachal Pradesh, Jammu & Kashmir, Uttaranchal (Saini et al. 2006); Pakistan: Islamabad (Togashi 1989). It is ambiguous whether these records actually base on *Tyloceridius dorsatus* or on the new *Tyloceridius stictocephalus*. We studied material from Himachal Pradesh, Jammu & Kasmir and Uttaranchal. Referring to Muche (1983), Saini (2007) recorded *Tyloceridius dorsatus* also from Nepal and Bhutan, but Muche mentioned only 1 ♀ (!) from India (“Chaurengi” located in former Uttarpradesh).

Lectotype (Fig. 11): 1♀, “Himalaya, Plasow”; “*Allantus dorsatus* Mocs., India Oriental”; “Lectotypus *Allantus dorsatus* Mocs., design. A. Taeger, 1988” [red]; “*Tyloceridius dorsatus* (Mocs.), det. A. Taeger, 88”. (HNHM). Left antenna, apical 3 antennomeres of right antenna, right foreleg below femur, left middle tarsus, right hind tarsus and left leg below femur are missing.

Paralectotype. 1 ♀, “Himalaya, Plasow”; “Typus *Allantus dorsatus* Mocs.”; “Syntype [sic!] *Allantus dorsatus* Mocsáry, 1883. teste A. Taeger, 2011” [red]; “*Tylocerus* [sic!] gen. n. *bituberculatus* Cam., Malaise det. 1935”; “DEI-GISHym 10877” (HNHM, figs see http://dx.doi.org/10.6084/m9.figshare.781292).

#### Other specimens examined.

1 ♀ (Figs 12–15), India, Uttaranchal (former N Uttar Pradesh), 5. 7. 1989, Rishikesh [30.117°N, 78.317°E], A. Riedel leg. (SDEI); 1♀ same data (see http://dx.doi.org/10.6084/m9.figshare.903712); 2 ♀♀, India, Kalatop, (H.P.), 8200’, 11.7.83, M.S. Saini (USNM); 1♀1♂, India, Himachal Pradesh, Kalatop, 2400m, July 1983, M.S. Saini collector, *Tyloceridius dorsatus* Malaise (!) (USNM); 1 ♀ 2 ♂♂, kept in NHRS, data unrecorded; 1 ♀ 1 ♂ India, Uttarakhand, Joshimath, 14.6.1983, leg. Balbir (NHRS, photo documentation by H. Vårdal).

#### Remarks.

Saini (2007) described both sexes and illustrated the lancet, penis valve and gonoforceps. This species is widely distributed at higher altitudes in Uttaranchal & Himachal Pradesh (Saini 2007).

## Discussion

The two species of *Tyloceridius* are very similar, and as *Tyloceridius stictocephalus* seems to be also rather variable in sculpture and color, the species are to be identified by consideration of the character sets given in the key. The genitalia of the taxa are not suitable for identification. According to the present data, *Tyloceridius dorsata* seems to be a species from the western Himalayas, whereas *Tyloceridius stictocephalus* is hitherto only known from the central Himalayas.

The type of *Rhogogastera bituberculata* Cameron, 1906 could not be found in the course of the present study. Cameron’s description disagrees in several aspects with *Tyloceridius dorsatus* and *Tyloceridius stictocephalus*. But as Malaise (1945) synonymized the taxon after examination of the types of *Allantus dorsatus* and *Rhogogastera bituberculata*, and we could not find any other specimens that fit with Cameron’s description, we assume that Malaise’s synonymization is correct. This is supported also by the type locality (Kashmir, western Himalayas).

Goulet (1996) considered *Tyloceridius* to be the sister group of *Rhogogaster* Konow, 1884. This is based on three shared derived characters (green color, not expressed submarginal furrow of the pronotum, and very narrow apical lobe of the metepimeron). Currently, there is no additional evidence to support this hypothesis. The only available COI barcode for *Tyloceridius* (DEI-GISHym 17228, BOLD:ACG2198) is rather distant (about 10 %) from its nearest neighbors *Tenthredo aaliensis* (Strand, 1898) and *Tenthredo xanthoptera* Cameron, 1876), while the distance to *Rhogogaster* species is about 13–14 %.

## Supplementary Material

XML Treatment for Tyloceridius

XML Treatment for Tyloceridius stictocephalus

XML Treatment for Tyloceridius dorsatus
